# 吉非替尼治疗127例晚期复发非小细胞肺癌患者分类及回归树分析

**DOI:** 10.3779/j.issn.1009-3419.2011.09.04

**Published:** 2011-09-20

**Authors:** 子平 王, 继红 郭, 燕 王, 雨桃 刘, 娟 杨

**Affiliations:** 1 100021 北京，中国医学科学院北京协和医学院肿瘤医院内科 Department of Medical Oncology, Cancer Hospital & Institute, Academy of Medical Sciences and Peking Union Medical College, Beijing 100021, China; 2 610041 成都，四川大学华西公共卫生学院流行病教研室 Department of Epidemiology, West China School of Public Health Sichuan University, Chengdu 610041, China

**Keywords:** 肺肿瘤, 吉非替尼, 分类及回归树, Lung neoplasms, Gefitinib, Classification and regression tree (CART)

## Abstract

**背景与目的:**

二线或三线使用吉非替尼治疗化疗后失败的非选择性晚期非小细胞肺癌（non-small cell lung cancer, NSCLC）的近期疗效只有10%-20%，女性、不吸烟、腺癌及亚裔具有更多的生存优势。然而临床中很难遇到符合以上所有条件的患者，所以有必要在临床中探索一些新的可以预测吉非替尼二、三线治疗晚期NSCLC生存时间的因素以及这些因素之间的相互影响。

**方法:**

对2005年3月-2010年3月在中国医学科学院肿瘤医院使用吉非替尼治疗的晚期NSCLC的临床资料和生存资料采用分类及回归树（classification and regression tree, CART）分析。

**结果:**

127例患者的中位无肿瘤进展生存时间（progression-free survival, PFS）为8个月（95%CI: 5.8-10.2）。CART分析将一线化疗疗效及年龄分别作为第一级及次级划分位点，逐级获得3个终末亚组。生存时间最短的是一线化疗进展（progressive disease, PD）的患者，中位PFS仅为1个月（95%CI: 0.8-1.2），处于中间位置的为一线化疗中取得部分缓解（partial response, PR）或稳定（stable disease, SD）的患者，年龄 < 70岁患者的中位PFS为10个月（95%CI: 7.0-13.0），而生存时间最长患者的中位PFS为22.0个月（95%CI: 3.8-40.1），为一线化疗后PR或SD且年龄≥70岁的患者。

**结论:**

一线化疗后PR或SD且年龄≥70岁的患者可以获得较长的生存时间，而化疗后进展的患者生存时间不佳。回归树分析可以找出既往被忽略的亚组患者，这对临床工作具有重要的指导意义，并将有利于今后开展相关的临床研究。

## Abstract (en)

**Background and objective:**

It has been proven that gefitinib produces only 10%-20% tumor regression in heavily pretreated, unselected non-small cell lung cancer (NSCLC) patients as the second- and third-line setting. Asian, female, nonsmokers and adenocarcinoma are favorable factors; however, it is difficult to find a patient satisfying all the above clinical characteristics. The aim of this study is to identify novel predicting factors, and to explore the interactions between clinical variables and their impact on the survival of Chinese patients with advanced NSCLC who were heavily treated with gefitinib in the second- or third-line setting.

**Methods:**

The clinical and follow-up data of 127 advanced NSCLC patients referred to the Cancer Hospital & Institute, Chinese Academy of Medical Sciences from March 2005 to March 2010 were analyzed. Multivariate analysis of progression-free survival (PFS) was performed using recursive partitioning, which is referred to as the classification and regression tree (CART) analysis.

**Results:**

The median PFS of 127 eligible consecutive advanced NSCLC patients was 8.0 months (95%CI: 5.8-10.2). CART was performed with an initial split on first-line chemotherapy outcomes and a second split on patients' age. Three terminal subgroups were formed. The median PFS of the three subsets ranged from 1.0 month (95%CI: 0.8-1.2) for those with progressive disease outcome after the first-line chemotherapy subgroup, 10 months (95%CI: 7.0-13.0) in patients with a partial response or stable disease in first-line chemotherapy and age < 70, and 22.0 months for patients obtaining a partial response or stable disease in first-line chemotherapy at age 70-81 (95%CI: 3.8-40.1).

**Conclusion:**

Partial response, stable disease in first-line chemotherapy and age ≥ 70 are closely correlated with long-term survival treated by gefitinib as a second- or third-line setting in advanced NSCLC. CART can be used to identify previously unappreciated patient subsets and is a useful method for dissecting complex clinical situations. Moreover, CART can be used to identify homogeneous patient populations in clinical practice and future clinical trials.

非小细胞肺癌（non-small cell lung cancer, NSCLC）是死亡率最高的恶性肿瘤，应用第三代含铂类化疗方案治疗的中位生存时间为9.5个月-10.5个月，1年生存率约40%^[1]^。晚期NSCLC几乎不可治愈，化疗药物的疗效已经达到平台，很难进一步提高患者的生存时间。近年来，表皮生长因子受体酪氨酸激酶抑制剂（epidermal growth factor receptor-tyrosine kinase inhibitors, EGFR-TKIs）的出现（如吉非替尼、厄洛替尼）明显延长了NSCLC患者的生存时间。小分子药物EGFR-TKIs可以结合到EGFR膜内的ATP结合区从而抑制信号传导，达到抑制肿瘤细胞生长的作用，是治疗NSCLC的新途径，使疗效达到了新的水平。对化疗失败的NSCLC使用吉非替尼治疗后缓解率约为10%-20%，且不良反应较轻，主要为皮疹和腹泻^[2]^。研究^[3-6]^发现二线或三线治疗对某些患者临床获益更加明显，如女性、无吸烟史、亚裔、腺癌或皮肤毒性等。

尽管目前的研究^[7]^已经发现*EGFR*突变对于一线使用吉非替尼治疗晚期NSCLC具有决定性的预测价值，缓解率高达71%。然而，TKIs在二线及三线治疗的疗效与*EGFR*基因突变的关系尚处于探讨中。临床实践中当患者同时具有有利及不利因素时，临床医生难以决定是否给予患者靶向治疗。针对这种临床困惑我们对中国医学科学院肿瘤医院晚期NSCLC患者二、三线使用吉非替尼的资料进行了回顾性分析。

## 1 资料与方法

### 1.1 纳入标准

① 有细胞学或病理学证据，按照IASLC第7版NSCLC TNM临床分期标准明确分期的晚期NSCLC（Ⅲb期、Ⅳ期）；②曾经至少接受过1个疗程化疗，且根据RECIST标准进行客观疗效评估；③二线及三线使用吉非替尼（商品名：易瑞沙）治疗，临床及生存资料完整。

### 1.2 排除标准

① 细胞学或病理学、临床分期不明或欠缺的病例；②三线以后使用吉非替尼治疗的病例；③曾经患有其它肿瘤。

### 1.3 临床资料

对2005年3月-2010年3月中国医学科学院肿瘤医院127例符合以上研究条件的病例资料使用SPSS 15.0软件进行统计分析。

### 1.4 统计学方法

以开始口服吉非替尼至有证据显示病情进展或死亡之间的时间间隔作为无肿瘤进展生存时间（progression-free survival, PFS）。未登记死亡日期的患者，以病历记录的最后日期代替死亡日期，并且在生存分析中按照删失数据处理。采用*Kaplan-Meier*法描述生存时间的分布情况。本研究以PFS为因变量，性别、年龄、吸烟状况、组织学类型、临床分期、一线化疗药物类型（含铂及非铂类方案）、一线化疗疗效、受累器官数目及器官转移部位、吉非替尼使用时机（二线或三线）等临床参数为自变量进行分类及回归树（classification and regression tree, CART）分析。横向有效分层取值为10，最大深度为3，选择母节点20，子节点8。在进行CART分析时，前述删失数据按照完整数据处理。生存树生长过程中，子结的划分采用杂质缩减最大化的基本思路，对众多自变量进行比较，并筛选出最佳分类变量和最佳分类结果，树的修剪采用成本-复杂度最小原则。

## 2 结果

### 2.1 晚期NSCLC患者临床资料

共127例晚期NSCLC病例符合以上条件，中位年龄56岁（20岁-81岁）。男性81例（63.8%），女性46例（36.2%）。 < 70岁111例（87.4%），≥70岁16例（12.6%）。具有1、2、3、≥4个脏器转移的病例数分别为47例（37.0%）、47例（37.0%）、25例（19.7%）及8例（6.3%）。转移部位最多的为胸膜77例（60.6%），其次为肺76例（59.8%）、骨骼39例（30.7%）、脑30例（23.6%）、肾上腺及肝脏均为9例（7.1%）、其它部位7例（5.5%）。组织学类型包括腺癌58例（45.7%）[高分化腺癌28例（22.0%）、中低分化腺癌30例（23.6%）]、鳞癌3例（2.4%）、肺泡癌2例（1.6%）、其它6例（4.7%）。Ⅲb期4例（3.1%），Ⅳ期123例（96.9%）；一线化疗曾经使用含铂类化疗方案的119例（93.7%），非铂类方案8例（6.3%）；一线化疗后疗效达部分缓解（partial response, PR）38例（29.9%）、稳定（stable disease, SD）63例（49.6%）、进展（progressive disease, PD）26例（20.5%）；二线及三线使用吉非替尼的分别有76例（59.8%）及51例（40.2%）。

### 2.2 生存情况

127例晚期NSCLC患者中，失访2例，其余生存资料完整。中位PFS为8.0个月（95%CI: 5.8-10.2），*Kaplan-Meier*生存曲线见图 1。

**1:**
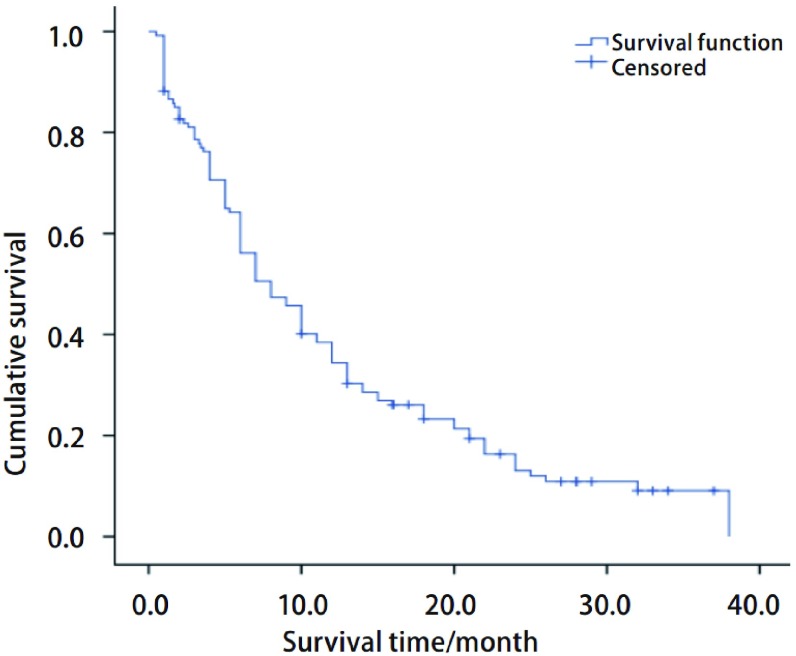
127例晚期NSCLC患者PFS生存曲线。中位PFS为8个月（95%CI: 5.8-10.2）。 *Kaplan*-*Meier* survival curve of 127 cases of advanced non-small cell lung cancer (NSCLC). The median progression-free survival (PFS) was 8.0 months(95%CI: 5.8-10.2).

### 2.3 CART分析结果

经统计学软件计算后自动生成各个划分位点。CART分析将一线化疗疗效作为第1个划分位点，年龄作为第2个划分位点年龄（≥70岁*vs* < 70岁）。逐级划分后获得了3个末端结，即3个终末亚组，分别是第2、3、4亚组（图 2）。生存时间最长的亚组是第4亚组，为一线化疗达PR或SD且年龄≥70岁的病例，中位PFS达22个月。其次为第3组，一线化疗PR或SD且年龄为 < 70岁的病例，中位PFS达10个月。生存时间最短的亚组是第2亚组，即一线化疗后PD的病例，中位PFS仅为1个月。一线化疗疗效、年龄与PFS关系密切，一线化疗达PR、SD的患者PFS长于化疗后进展病例，并且≥70岁病例的生存时间明显长于 < 70岁病例。3个亚组生存曲线见图 3。

**2:**
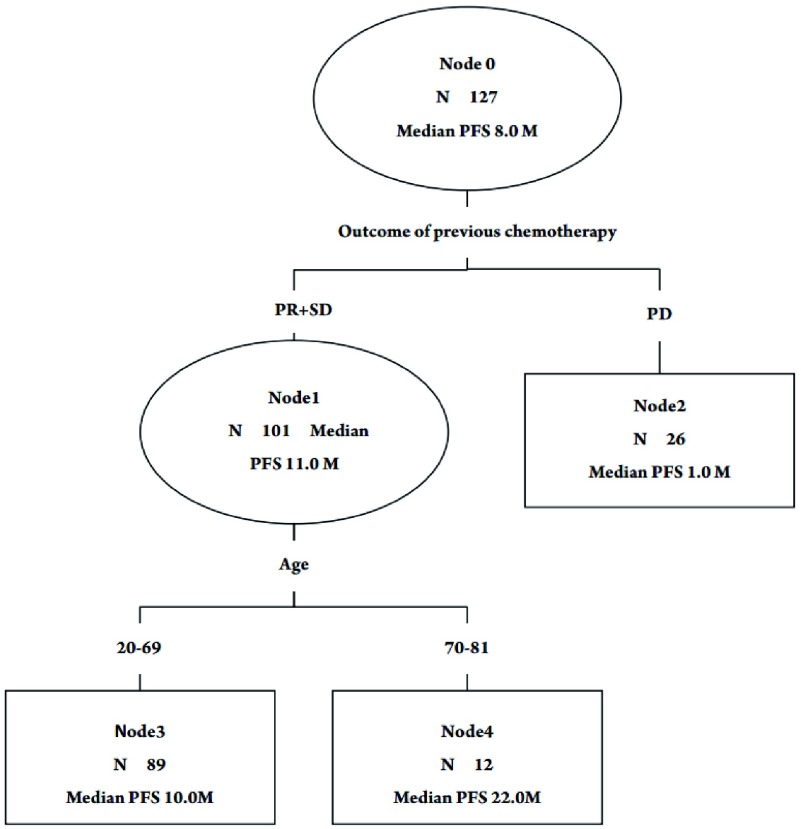
分类及回归树图形，首个划分位点为化疗疗效，次级划分位点为年龄。 CART generated with the initial split on the outcome of previous chemotherapy, and then, on the age of patients.

**3:**
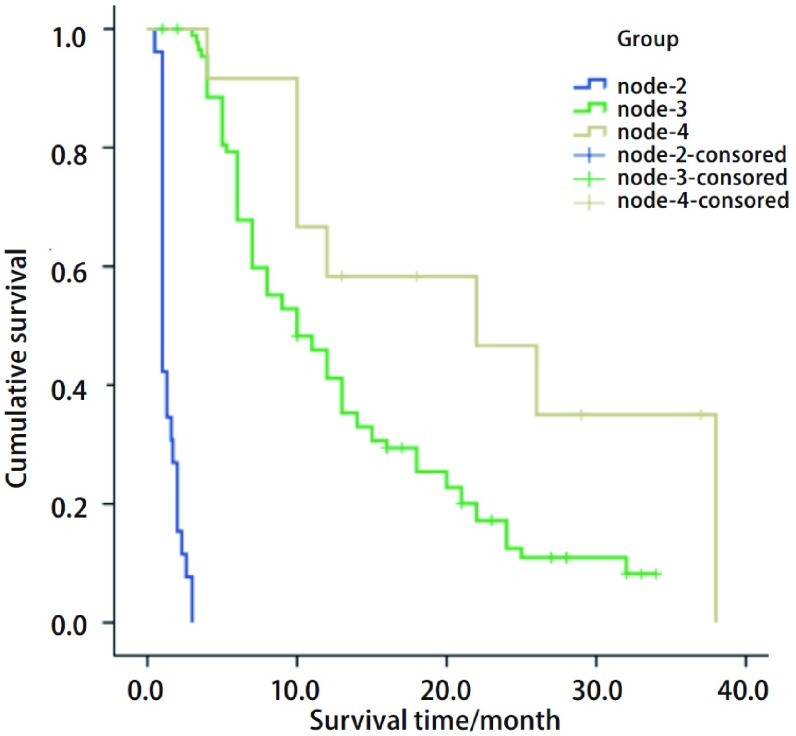
CART分析后的3个终末亚组的PFS生存曲线，第2、3、4组的中位PFS分别为1个月、10个月、22个月。 *Kaplan*-*Meier* survival curves of the 3 terminal subgroups generated from the CART analysis. Median PFS of the second, third, and fourth subgroup is 1, 10, 22 months respectively.

## 3 讨论

吉非替尼是第一个用于治疗肺癌的分子靶向药物，属EGFR-TKIs，可明显延长化疗失败的晚期NSCLC患者的生存。既往研究的亚组分析发现具有某些临床特征的患者具有更多的生存机会。ISEL研究^[5]^显示虽然在二、三线治疗中吉非替尼与安慰剂相比略显生存优势，但无统计学差异，但在非吸烟及亚裔患者中具有生存差异。Paez等^[8]^发现另一些临床病理学特征与使用TKIs疗效相关，如日裔、女性、腺癌。Lynch等^[9]^首先揭示了*EGFR*基因突变与TKIs疗效之间的密切关系，随后一些研究也显示TKI疗效较好的临床指标与*EGFR*基因突变相关，如吸烟与否和*EGFR*基因突变呈明显的相关性，不吸烟与较少吸烟患者具有较高的突变比例^[10]^，甚至提示可以将这些临床因素看成是*EGFR*基因突变的替代指标^[11]^。

2009年IPASS研究^[7]^显示吉非替尼一线治疗具有*EGFR*基因突变的晚期NSCLC疗效较紫杉醇/卡铂方案突出，PFS具有明显优势。其它相关研究也得到了一致的结果。2010年INTEREST研究^[12]^的亚组分析发现具有*EGFR*基因敏感性突变的患者可以预测患者使用吉非替尼治疗的PFS。二、三线治疗的优势人群间接提示*EGFR*基因突变对药物的选择仍起着深刻的影响。

一些研究^[13]^显示非优势人群中也存在基因突变，曾经或正在吸烟的患者其*EGFR*基因突变比例为23%。所以，根据某一项非优势或优势指标就拒绝或接受使用吉非替尼有可能出现忽略或夸大正确使用TKIs的人群。其次，具备所有优势因素的患者是很少的，更多患者同时具备优势和劣势因素，很难将几个因素结合起来综合预测患者的预后，例如是否应采用EGFR-TKIs二线治疗女性、腺癌、吸烟的晚期NSCLC患者。

已有研究^[14]^发现结合多个基因较单基因更能准确预测TKIs的疗效。临床中依靠单个优势因素预测往往可能夸大事实，故有必要进行多因素的研究。常用的进行生存时间研究的多因素分析方法，如多元线性回归分析、*Logistic*回归、*Cox*回归等，多要求样本来自同一总体，具有同质性。但许多临床资料很难满足这一条件，因为同种疾病的患者，其内部同质性通常较差。CART分析不受上述条件的约束，可以将不同特征的个体分配到回归树的各个局部去处理，使每个局部样本的同质性得到改善，分类的特征变量即是某个局部样本的共同特点。CART的树型结构与临床思维十分接近，可体现生存特征，通过分级树的级数可阐明各研究变量的重要性。

因为靶向治疗后的治疗可以影响患者总生存时间，所以目前多采用PFS作为靶向治疗研究的终点指标以排除其后治疗的影响，本研究也采用PFS作为观察指标。化疗失败后127例NSCLC患者二线或三线使用吉非替尼治疗的中位PFS为8个月，较好的一线化疗疗效（PR或SD）与年龄≥70岁是长期生存的影响因素。有研究^[15]^显示*EGFR*基因突变与*ERCC1*基因低表达相关，ERCC1低表达又与铂类药物疗效正相关。本研究显示化疗疗效为PR或SD的患者较PD的患者具有更佳的生存优势，即化疗后进展的患者在吉非替尼治疗中也未获得更多的机会，可能反映了这种关系。IPASS研究分析中发现年龄≥65岁患者*EGFR*基因突变率为68.5%，而 < 65岁者为56.7%，亚组分析中发现这两组患者的PFS有差异（*P*=0.025, 6），所以年龄差异带来*EGFR*突变差异可能影响了患者的长期生存。本研究经计算后将年龄的分界限定为70岁，这与IPASS的研究结果相似，反映了可能的*EGFR*基因突变事件的差异。在本研究中，由于临床中过多选择了非吸烟或轻度吸烟的女性腺癌患者，非吸烟、腺癌、女性因素并没有在回归树分析中出现。我们的资料显示女性、不吸烟或少吸烟、腺癌的患者比例很高，在优势人群占主导地位时难以再将这些因素显现出来，但也有可能是多因素分析的结果，这些优势人群多存在于生存时间较长的亚组之中，样本扩大后将有所体现；此外，样本量进一步扩大后可能还会出现更多的影响因素，甚至是以往忽略的因素。使用TKIs后生存获益最多人群的组织标本资料将会为基础研究提供宝贵的信息，深入的分析和了解将会对二线及以后的治疗提供更加有力的依据。

目前*EGFR*基因突变与二、三线使用TKIs治疗疗效的关系尚在探讨之中，临床工作中根据临床特征间接预测可能敏感的患者非常重要。通过采用CART分析方法通过对127例NSCLC患者临床信息的分析，本研究发现一线化疗疗效、年龄是影响二、三线使用吉非替尼治疗晚期NSCLC患者生存的重要因素，但该结果尚需大样本研究做进一步验证。
